# Design, methods and demographic findings of the DEMINVALL survey: a population-based study of Dementia in Valladolid, Northwestern Spain

**DOI:** 10.1186/1471-2377-12-86

**Published:** 2012-08-30

**Authors:** Miguel Angel Tola-Arribas, María José Garea, María Isabel Yugueros, Fernando Ortega-Valín, Ana Cerón, Beatriz Fernández-Malvido, Marta González-Touya, Antonio San José, Ana Botrán, Vanessa Iglesias, Bárbara Díaz-Gómez

**Affiliations:** 1 Department of Neurology, Hospital Universitario Río Hortega, Valladolid, 47012, Spain; 2 Department of Geriatrics, Hospital Universitario Río Hortega, Valladolid, Spain; 3 Department of Psychology, Hospital Universitario Río Hortega, Valladolid, Spain; 4 Family Physician, Centro de Salud Campo Grande, Valladolid, Spain; 5 Family Physician, Centro de Salud Parquesol, Valladolid, Spain

**Keywords:** Dementia prevalence, Epidemiology, Undiagnosed dementia, Population-based survey, Seven-minute screen, Anosognosia, Nutritional assessment

## Abstract

**Background:**

This article describes the rationale and design of a population-based survey of dementia in Valladolid (northwestern Spain). The main aim of the study was to assess the epidemiology of dementia and its subtypes. Prevalence of anosognosia in dementia patients, nutritional status, diet characteristics, and determinants of non-diagnosed dementia in the community were studied. The main sociodemographic, educational, and general health status characteristics of the study population are described.

**Methods:**

Cross-over and cohort, population-based study. A two-phase door-to-door study was performed. Both urban and rural environments were included. In phase 1 (February 2009 – February 2010) 28 trained physicians examined a population of 2,989 subjects (age: ≥ 65 years). The seven-minute screen neurocognitive battery was used. In phase 2 (May 2009 – May 2010) 4 neurologists, 1 geriatrician, and 3 neuropsychologists confirmed the diagnosis of dementia and subtype in patients screened positive by a structured neurological evaluation. Specific instruments to assess anosognosia, the nutritional status and diet characteristics were used. Of the initial sample, 2,170 subjects were evaluated (57% female, mean age 76.5 ± 7.8, 5.2% institutionalized), whose characteristics are described. 227 persons were excluded for various reasons. Among those eligible were 592 non-responders. The attrition bias of non-responders was lower in rural areas. 241 screened positive (11.1%).

**Discussion:**

The survey will explore some clinical, social and health related life-style variables of dementia. The population size and the diversification of social and educational backgrounds will contribute to a better knowledge of dementia in our environment.

## Background

Demographic changes in world population during the next decades will cause an appreciable increase in the number of dementia patients [1]. Faced with the absence of disease-modifying treatments, control of dementia risk factors will be essential. A 10% reduction in diabetes, hypertension, obesity, smoking, depression, and cognitive or physical inactivity could potentially prevent more than 1 million cases of dementia worldwide [2]. More studies are required of the prevalence and incidence of dementia to chart the course of the disease, to confirm a cautious optimism in epidemiological trends [3], and to allow policymakers to plan the need for services.

In recent years several dementia prevalence surveys have been carried out in Spain [4-7] or are in progress [8] with considerable geographic variation that may be method-related [4]. A detailed analysis of the etiological subtypes, beyond Alzheimer’s disease (AD) and vascular dementia, has been generally lacking. To our knowledge, the prevalence of anosognosia, which is very high in memory clinics [9], has never been evaluated in population studies. Other health related life style variables such as nutrition or factors associated with the lack of or the delay in the diagnosis of the disease in the community [10,11] have barely been analyzed and thus justify new research in this field.

In 2008 we initiated the Dementia in Valladolid (DEMINVALL) survey in two geographically well-defined urban and rural populations in northwestern Spain aimed at describing (1) the prevalence and incidence of dementia and its subtypes, (2) the prevalence of anosognosia in dementia patients, (3) the characteristics of undiagnosed dementia in the community, and (4) the nutritional status and the daily consumption of micro and macronutrients. In this report we describe the design, methodology, and main general health, educational, and sociodemographic features of the study population.

## Methods/Design

### Geographical area

In January 2009, the population of the province of Valladolid was 532,575 (18.2% ≥ 65 years) with 6.7% of institutionalized persons. The city of Valladolid (189 Km northwest of Madrid) has a population of 317,864, which has remained stable during last 10 years [12]. The main economic activities are manufacturing and service sector in urban areas and agriculture and viticulture in rural ones. Health care in Valladolid is organized into two separate areas, each with a university hospital that includes a neurology department and a geriatrics unit. Primary care is provided at community health centers and in doctors’ offices in small rural areas.

### Study population

The participants were sampled from the primary care registry of Social Security health card holders. In Spain, health care is free and universal, covering virtually the entire population. This selection method has various advantages: (1) it is continually updated; (2) provides personal data such as address, phone number and primary care physician; (3) provides access to medical record; (4) improved external validity.

In order to obtain a sample of all educational backgrounds and social classes in Valladolid, in addition to a good diversification of dementia risk factors, a mixed population -urban and rural- was selected. Community-dwelling and nursing home residents were included.

#### Rural residential environment

**Figure 1:**
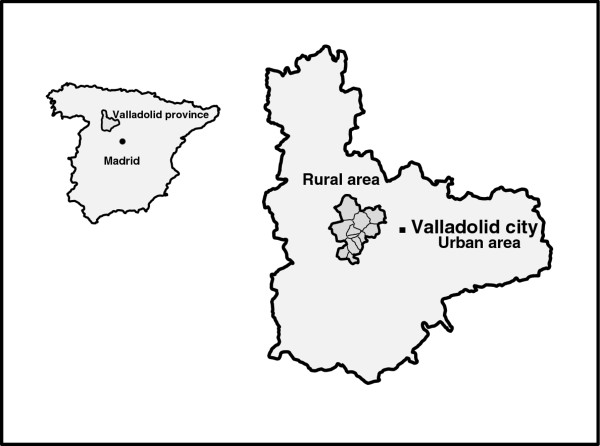
Location of the province of Valladolid and the rural area (municipalities of Villanubla, Peñaflor de Hornija, Wamba, Ciguñuela, Castrodeza, Torrelobatón, Matilla de los Caños, Robladillo, Villán de Tordesillas, Velilla and Velliza).

We selected the whole population aged 65 years and over of 11 municipalities of the “Montes Torozos” region (870 residents) in northwestern Valladolid (338.9 Km^2^, population density 12.6/km^2^). In this rural area (Figure 1), primary care is available in two community health centers. The population is mainly blue-collar workers.

#### Urban residential environment

Residents aged 65 years and over from the districts of Campo Grande and Parquesol of the city of Valladolid (6,183 individuals) were selected. Each of these districts has a community health center. Due to the large population size, the survey was based on a 34.2%, random, 5-year, age- and sex-stratified sample comprising 2,119 individuals. The size was calculated in order to provide an estimated 6.5% prevalence, 1% precision, 95% confidence interval, and 20% predicted losses. Both white and blue-collar workers are represented in this sample.

The eligibility criteria for this mixed population of 2,989 subjects were age 65 years and over on 1 February 2009 and at least 6 months residence in the previous year in the selected geographic areas. These areas were chosen for the following reasons: (1) existence of a computerized registry of the medical diagnoses of the participants; (2) the Rio Hortega University Hospital (RHUH) is the reference centre for the neurological and geriatric evaluation for both areas, with a single neurological team and a geriatrics unit; (3) a close relationship between the researchers and the primary care physicians; (4) proximity of the rural area to the RHUH.

### Study design

DEMINVALL is a cross-over and cohort, population-based study. A cross-sectional, two-phase door-to-door design was adopted. Point prevalence was used as a disease frequency measure. The prevalence date was 1 February 2009. In order to be included in the prevalence numerator the patients had to be alive and had to meet the diagnostic criteria for dementia on or before this date.

The patients detected in the prevalence study have been followed up at regular intervals in the out-patient clinics of neurology and geriatrics in the RHUH. A dementia-free cohort was generated. The survivors of this cohort will be re-evaluated between October 2012 and May 2013 to describe the incidence of dementia and subtypes following a minimum observation period of three years from the prevalence date. A similar methodology will be used in two phases.

### Phase 1: screening

In October 2008, a meeting of the researchers was held in which different clinical and epidemiological aspects of dementia were reviewed. Prior to the beginning of the survey a letter was sent to all selected subjects inviting them to participate and stating the absence of risk. In addition, the study was announced on the local radio, television, and press, and posters were placed in the health centers in order to better publicize the study.

#### Field work team

The participants were evaluated by 27 primary care physicians and one geriatrician from the largest nursing home. Five of the physicians coordinated the field work. All were trained to standardize the answers and to interpret the screening instruments. Whenever possible, the evaluation was carried out by the regular physicians.

#### Screening protocol

The initial interview was arranged by telephone and was held in the community health centers, physician’s offices, or when necessary, in the participant’s home or nursing home. At least five attempts to locate a potential participant within a period of three months were required in order to classify a subject as a non-located. Participants were asked to provide their medical reports and medications. Available medical records were also reviewed. The duration of the evaluation was 30 minutes and, when necessary, it was carried out in the presence of reliable informants. The evaluation period of the first phase was from February 2009 to February 2010.

Before administering the screening test, the eligibility criteria were confirmed and a structured questionnaire was filled out. Data concerning marital status, cohabitation, educational level and occupation history were collected. Social status was calculated according to an algorithm based on profession and level of training [13]. If a previous diagnosis of dementia existed, the type, onset date, main initial symptom, and date and level of care of diagnosis were evaluated. All those patients previously diagnosed with dementia were evaluated in the second phase of the study, regardless of whether or not the screening test results were negative or positive. In the absence of this diagnosis, existence of prior consultations for cognitive impairment, complaint symptoms and level of care in which they were held were evaluated. Also, information relative to the subjective health status, family history of dementia, cranial trauma, thyroid disease, and depression was collected. History of Parkinson’s disease, essential tremor, stroke, ischemic heart disease, hypertension, and diabetes were evaluated. Duration of the illness, medications, and degree of control were specified. A history of tobacco and alcohol consumption (standard units of alcohol intake per week) was taken [14]. In addition, physical activity during the previous year was stratified according to intensity and to the number of days of exercise per week [15]. The blood pressure of those subjects without a history of hypertension was measured on two separate occasions. Finally, the body mass index was calculated.

#### Screening instruments

The 7 Minute Screen Neurocognitive Battery (7MS) was used [16]. This instrument evaluates temporal orientation, naming, delayed free- and cued-recall test of memory impairment, visuospatial organization, semantic processing and storage. A score of <20 points (8 percentile), regardless of age and cultural level, was considered positive. When it was not possible to perform the 7MS, the reasons were given and the short version of the Informant Questionnaire on Cognitive Decline in the Elderly (IQCODE) [17] was employed. A score of 57 and over was considered positive. In case of death after the prevalence date, collaboration of a close relative was sought, medical and sociocultural data were collected, and a Spanish version of the Kawas Dementia Questionnaire [18] was employed. This instrument provides a structured review of the general diagnostic criteria of dementia, AD, vascular dementia, and secondary dementia.

### Phase 2: Diagnostic confirmation

#### Clinical research team

The neurological assessment was carried out by four neurologists and one geriatrician, all experienced in dementia evaluation. Three neuropsychologists performed the neuropsychological evaluation. The coordinators of the first phase carried out the nutritional assessment, aided by the research unit of the RHUH and by a dietician. Two social workers conducted the social evaluation by telephone. A nurse took blood samples in the homes of those participants unable to travel. Genomic DNA was extracted from whole-blood samples and amplified by using polymerase chain reaction in the genetic department of the RHUH to determine ApoE genotype.

#### Assessment protocol

A comprehensive neurological assessment was offered to those participants with a positive screen or previous diagnosis of dementia. In addition, a randomly selected control group of 160 subjects was included (8.3% of those who screened negative). The proportions of sex and age stratification with regard to those who screened positive were respected. The assessment was carried out in the out-patient clinics of neurology, geriatrics, and clinical psychology in the RHUH during two or three visits in the presence of a reliable informant. It was performed in the subject’s home when necessary. The evaluation period was from May 2009 to May 2010. After revising the medical record and medications, a structured neurological examination was performed.

A brain imaging test (cranial CT or 1.5 T MRI) was suggested for all the subjects who screened positive. A single photon emission computed tomography imaging using [123] FP-CIT (DaTSCAN; GE Healthcare) was carried out on some patients with dementia and parkinsonism. Complete blood count, biochemical analysis, lipids, vitamin B_12_ levels, thyroid function, luetic serology, homocysteine, albumin, prealbumin, transferrin, and ApoE genotype were analyzed.

The neuropsychologic evaluation consisted of the use of the Cambridge Examination of Mental Disorders of the Elderly (CAMDEX) [19]. This instrument includes the necessary information to diagnose dementia and its subtypes, and includes an interview with an informant, the Blessed Scale, and an ischemic and depression scales. It also includes the Cambridge Cognitive Examination (CAMCOG) [20] that permits the scoring of the Mini-Mental State Examination (MMSE).

In addition, the protocol included the following instruments: (1) Rapid Disability Rating Scale-2 [21]; (2) Pittsburgh Sleep Quality Index [22]; (3) Zarit scale for assessing caregiver burden [23]; (4) Clinical Dementia Rating (CDR) [24]; (5) Global Deterioration Scale for assessment of primary degenerative dementia (GDS) [25], and (6) Functional Assessment Staging (FAST) [26]. In order to evaluate anosognosia, the Clinical Insight Rating Scale [27] and the Rating of Awareness Deficit [28], which were previously validated, were applied. The consumption of healthcare resources during the previous year was quantified by employing a questionnaire based on a national health survey of the Spanish National Institute of Statistics [29]. For the social evaluation, a structured interview of widespread use in Spain was employed [30].

For the nutritional evaluation, the Mini Nutritional Assessment Test was used [31]. In addition, a nutritional questionnaire was carried out in order to calculate the daily consumption of macro and micronutrients. To this end subjects were given a form in which they registered their consumption during a two-day period, one of the days falling on a weekend. The dietary data was managed by means of a personal computer, and the use of food scales and models to enhance portion size accuracy were incorporated. Records were reviewed by a registered dietitian and analyzed with a computer-based data evaluation system. National composition food tables were used as a reference [32].

#### Diagnostic criteria

Clinical diagnosis of dementia was established by agreement between the neurologist or geriatrician and the neuropsychologist responsible for each individual evaluation. Problematic cases and all the dementia questionnaires were reviewed by a second neurologist (M.A.T-A.).

The criteria of the Diagnostic and Statistical Manual of Mental Disorders (DSM-IV) [33] were applied for the diagnosis of dementia syndrome. In addition, the criteria of NINCDS-ADRDA for AD [34], the criteria of NINDS-AIREN for vascular dementia [35], the criteria of the DLB Consortium for dementia with Lewy bodies [36], the criteria for dementia associated with Parkinson’s disease [37], and the criteria for frontotemporal lobar degeneration were applied [38]. AD with cerebrovascular disease was regarded as present in those patients that met the criteria of NINCDS-ADRDA for possible AD and who presented significant levels of small vessel ischemic changes, strategic lacunar infarcts, or large vessel infarcts on brain imaging and, in addition, who had a history of stroke or impaired neurological examination (focal deficit or gait disturbances) [39]. Secondary dementia was classified as having an identifiable or probable cause. When the clinical information was insufficient to reach an etiologic classification, undetermined dementia was diagnosed.

### Ethical aspects

The survey was approved by the Ethics and Clinical Research Committee of the RHUH. All participants (or a relative when necessary) were asked to sign an informed consent. The database was inscribed in the Spanish Agency for Data Protection. Treatment and follow-up were offered to all patients with undetected dementia. All other non-neurological processes detected were reported to the primary care physicians.

## Results

**Figure 2:**
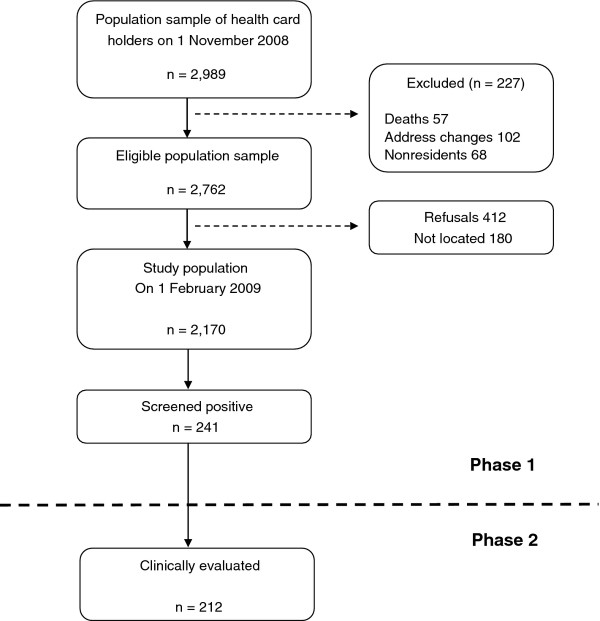
**Flow chart showing the population selected and finally evaluated**.

Figure 2 shows a flow chart of participant attrition. 227 subjects (7.6%) were excluded from the initial population for different reasons. Of the 2,762 eligible subjects, 180 (6.5%) were non-located and 412 (14.9%) refused to participate. The final population evaluated in the first phase was composed of 2,170 individuals; 241 (11.1%) screened positive. Table 1 shows the distribution of the final population by age, sex and area of residence. Table 2 displays the differences between this population, non-located and non-collaborators. A smaller proportion of non-responders in rural areas was statistically significant. We obtained information about the educational level of 2,115 participants (97.5%). Table 3 shows the distribution by age and gender. The sociodemographic data, occupation, general health, vascular risk factors and habits are displayed in Table 4, including the number of subjects analyzed in each case.

**Table 1:** Distribution of the evaluated population stratified by age, sex, and living environment

**Age, years**	**Urban population (n = 1,459)**		**Rural population (n = 711)**		**Total population (n = 2,170)**	
	**Men, n**	**Women, n**	**Men, n**	**Women, n**	**Men, n**	**Women, n**
65–69	162 (27.7%)	213 (24.4%)	91 (26.1%)	83 (22.9%)	253 (27.1%)	296 (23.9%)
70–74	154 (26.3%)	177 (20.3%)	84 (24.1%)	60 (16.5%)	238 (25.5%)	237 (19.2%)
75–79	124 (21.2%)	160 (18.3%)	79 (22.7%)	86 (23.7%)	203 (21.8%)	246 (19.9%)
80–84	83 (14.2%)	152 (17.4%)	61 (17.5%)	68 (18.7%)	144 (15.4%)	220 (17.8%)
85–89	42 (7.2%)	111 (12.7%)	26 (7.5%)	40 (11.0%)	68 (7.3%)	151 (12.2%)
≥90	20 (3.4%)	61 (7.0%)	7 (2.0%)	26 (7.2%)	27 (2.9%)	87 (7.0%)
**Total**	**585 (100%)**	**874 (100%)**	**348 (100%)**	**363 (100%)**	**933 (100%)**	**1,237 (100%)**

All percentages are column percentages.

**Table 2:** Differences among analyzed and non-located subjects and refusals

	**Analyzed**	**Non-located**	**Refusals**
	**n = 2,170**	**n = 180**	**n = 412**
Age (Mean ± SD), years	76.5 ± 7.8	76.6 ± 7.8	76.4 ± 7.9
Women (%)	57%	55%	61.2%
Institutionalized (%)	5.2%	2.2%	3.2%
Rural area (%)	32.8%	16.7%^1^	14.6%^2^
**Age, years**			
65–69	25.3%	28.9%	27.4%
70–74	21.8%	18.3%	19.2%
75–79	20.7%	16.7%	22.1%
80–84	16.8%	22.2%	16.0%
85–89	10.1%	8.9%	9.5%
≥90	5.3%	5.0%	5.8%

^1^ p < 0.001. Difference between non-located and analyzed subjects.

^2^ p < 0.001. Difference between refusals and analyzed subjects.

**Table 3:** Educational level of DEMINVALL participants by age groups and gender

**Age, years**	**Illiterate (n = 25)**		**Less than primary school (n = 451)**		**Primary school (n = 1,092)**		**Secondary school and higher (n = 547)**	
	**Men**	**Women**	**Men**	**Women**	**Men**	**Women**	**Men**	**Women**
65–69	2	2	41	49	104	165	104	79
70–74	0	2	31	43	129	139	78	47
75–79	1	6	46	64	93	124	61	47
80–84	1	6	39	59	69	107	28	41
85–89	1	3	13	40	37	73	13	28
≥90	0	1	6	20	11	41	6	15
**Total**	**5**	**20**	**176**	**275**	**443**	**649**	**290**	**257**

Total years of schooling: 9.0 ± 4.1.

**Table 4:** Characteristics of the evaluated population

**Sociodemographic**	
**Marital Status,% (n = 2,122)**	
Single	11.3
Married	60.1
Widowed	27
Separated/divorced	1.6
**Living arrangements,% (n = 2,170)**	
Alone	18.1
With spouse	45.7
With relatives	26.6
Institutionalized	5.2
Others (including rotation among relatives)	4.4
**Social class,% (n = 2,121)**	
Low	16.4
Medium-low	52.5
Medium	22.3
Medium-high	7.9
High	0.9
**Occupation/employment**	
**Work history,% (n = 2,121)**	
Blue-collar	67.2
White-collar	18.3
Services	12.7
Never employed	1.8
**Current occupation,% (n = 2,116)**	
Retired with no activity	54.7
Active in habitual profession	36.7
Housewife/husband	8.6
**General data concerning health and habits**	
**Subjective state of health,% (n = 2,067)**	
Bad or very bad	7.4
Average	29.1
Good or very good	63.5
**Body mass index (n = 2,029)**	27.3 ± 4.2
**Vascular risk factors,% (n =2,170)**	
Diabetes mellitus	15.8
Hypertension	55.7
Ischemic heart disease	10.7
Stroke	6.3
Ever-smoker	38.2
**Alcohol consumption,% (n = 2,124)**	
Never drinker	57.8
Active drinker	34.7
Ex –drinker	7.5
Daily consumption of alcohol (gr ethanol)	15.8 ± 1.5
Active drinkers at risk^1^	7.3^2^
**Physical exercise,% (n = 2,170)**	
Sedentary	18.1
Low activity	21.6
Moderate activity	47.4
High activity	12.9

^1^ Daily consumption of ethanol over 30 gr.

^2^ Percentage of active drinkers.

## Discussion

From a methodological point of view, the DEMINVALL study presents some interesting differential characteristics with respect to other similar studies undertaken during the past few years [4-8]. First of all, the entire process of screening of the participants was carried out by trained medical personnel. In many cases this evaluation was performed by the subjects’ habitual physicians. This fact contributed to a good participation in the urban area and to an optimum participation in the rural one. A similar approach was employed in the NEDISA [8] survey in Salamanca, Spain, in which a participation result of over 95% was obtained.

A second differential aspect is the choice of the screening instrument. In the majority of the population-based surveys local versions of the MMSE have been employed [40]. The limitations of this test are well known, especially with regard to sensitivity and low positive predictive value in individuals with a low cultural level [41]. The 7MS was originally proposed as a screening test for AD, although it usefulness in other types of dementias has been demonstrated [42]. Sensitivity and specificity of the 7MS are high and normative data concerning the Spanish population are available [43]. Due to its higher performance in the evaluation of episodic memory, it can contribute to the reduction of cost of screening in population studies, since it optimizes the detection of AD [44]. The main limitations are the administration time, which is normally over ten minutes, the requirement that the subject make a drawing of a clock, which limits its administration in patients with serious physical or sensorial defects, and the calculation of the score. Its usefulness has been placed in doubt in large epidemiological studies for these reasons [45]. Our initial hypothesis was that, despite these limitations, its greater efficacy allowed us to carry out a more reliable screening and, above all, a more efficient one (only 11.1% of the evaluated subjects were screened positive) with a lower consumption of healthcare resources in the second phase. The analysis of the false negatives in the control group and its positive predictive value will permit its efficiency to be confirmed in a later analysis. In any case, the protocol established the application of the IQCODE, with a sensitivity similar to that of the MMSE [46], in those cases in which the application of the 7MS was not possible.

The survey included a specific evaluation of the characteristics of undiagnosed dementia in the community with the purpose of explaining the determinants of the delay or lack of identification of the disease in our environment. Among these, we analyzed the loss of insight, quite frequent in dementia, although it has never been evaluated in population-based studies. Finally, the nutritional evaluation of subjects with confirmed dementia and their comparison with the control group will provide valuable data concerning the influence of diet on dementia and of the latter on the nutritional state of the patients. In this sense, a favorable effect of the Mediterranean diet typical to our environment has been demonstrated [47].

A major limitation of the DEMINVALL survey, as in the majority of the observational studies with large populations, is the sample attrition bias. From the initial population, 7.6% of the selected subjects were excluded because they did not comply with the eligibility criteria, in part due to outdated health cards. Among the eligible subjects 21% were excluded from the denominator for the prevalence calculation because they were non-responders. In the analysis of differences among the evaluated subjects and non-responders, only a lower participation in the urban area was statistically significant. The sample size of the latter, it should be pointed out, was calculated based on a predicted 20% loss; therefore, the impact bias will be lower.

In summary, we have presented the design and methodology of the DEMINVALL survey. It will allow us a more accurate view of the frequency of dementia in our environment and the reasons for diagnosis delay or the lack of detection. Therefore, we can contribute to a more efficient planning of health and social resources and to the implementation of plans that will facilitate early diagnosis, an aspect of maximum importance in the moment in which modifying treatments of the disease are available.

## Abbreviations

AD: Alzheimer's disease; DEMINVALL: Dementia in Valladolid; RHUH: Rio Hortega University Hospital; 7MS: Seven-minute screen neurocognitive battery; IQCODE: Informant questionnaire on cognitive decline in the elderly; MMSE: Mini-mental state examination.

## Competing interests

The authors declare that they have no competing interest.

## Authors' contributions

Conception of the idea and design of the study: MAT-A, MJG, MIY, FO-V. Development of the protocol and general organization: MAT-A, BF-M, MG-T, AS, AB, VI and BD-G. Field work organization and nutritional assessment: MG-T, AS, AB, VI and BD-G. Neurological assessment: MAT-A, MJG, MIY, FO-V, AC. Neuropsycological assessment: BF-V. Writing of the manuscript: MAT-A, AC. All authors read and approved the final manuscript.

## Funding

The survey was funded by the Gerencia Regional de Salud de Castilla y León (Grant GRS/340/09) and by the Fundación General de la Universidad de Valladolid.

## Pre-publication history

The pre-publication history for this paper can be accessed here:

http://www.biomedcentral.com/1471-2377/12/86/prepub
